# Co-emergence Reinforcement and Its Relevance to Interoceptive Desensitization in Mindfulness and Therapies Aiming at Transdiagnostic Efficacy

**DOI:** 10.3389/fpsyg.2020.545945

**Published:** 2020-12-22

**Authors:** Bruno A. Cayoun, Alice G. Shires

**Affiliations:** ^1^MiCBT Institute, Hobart, TAS, Australia; ^2^Mindfulness Integrated Therapies and Research Clinic, University of Technology Sydney, Ultimo, NSW, Australia; ^3^School of Psychology, Faculty of Science, The University of Sydney, Sydney, NSW, Australia

**Keywords:** interoceptive desensitization, mindfulness, transdiagnostic, co-emergence model of reinforcement, mindfulness-integrated cognitive behavior therapy, MiCBT, CMR

## Abstract

Interoception, the ability to feel the body’s internal sensations, is an essential aspect of emotional experience. There is mounting evidence that interoception is impaired in common mental health disorders and that poor interoceptive awareness is a major contributor to emotional reactivity, calling for clinical interventions to address this deficit. The manuscript presents a comprehensive theoretical review, drawing on multidisciplinary findings to propose a metatheory of reinforcement mechanisms applicable across a wide range of disorders. We present a reconsideration of operant conditioning through the co-emergence model of reinforcement, which is a neurophenomenological account of the interaction between cognition and interoception, and its consequences on behavior. The model suggests that during memory processing, the retrieval of autobiographical memory (including maladaptive cognition) is dependent upon its co-emerging interoceptive cues occurring at the encoding, consolidation and reconsolidation stages. Accordingly, “interoceptive reinforcement” during emotional distress is a common factor to all emotional disorders and a major cause for relapse. We propose that interoceptive desensitization has transdiagnostic benefits, readily achievable through the cultivation of equanimity during mindfulness training and can be integrated in cognitive and behavioral interventions to permit a transdiagnostic applicability. We summarize the contributions of this approach into 10 specific and testable propositions.

## Introduction

Whereas exteroception refers to the sensory perception of non-self-referential stimuli, such as seeing, smelling and hearing the external environment, interoception is the perception of the body’s internal states through body sensations that one tends to associate with the sense of self (Seth and Critchley, 2013). Interoception is now recognized to be a fundamental mechanism underlying motivation, emotion, social cognition and self-awareness (Tsakiris and Critchley, 2016), and interoceptive awareness is associated with individual differences in one’s ability to inhibit behavior (Rae et al., 2019). There is also increasing multidisciplinary evidence that interoception is predictably impaired in mental health conditions and should be considered as a transdiagnostic therapeutic target (Khalsa et al., 2018), especially given that psychiatric patients do not completely remit and at least 50% do not respond to standard treatments (Leichsenring et al., 2019). Accordingly, therapy approaches that emphasize interoceptive mechanisms are important to consider and are likely to be central to future developments in clinical interventions.

Interoceptive awareness and acceptance are central skills in mindfulness meditation (Hart, 1987; Cayoun, 2011; Bornemann et al., 2015; Hanley et al., 2017), and there is evidence supporting the efficacy of mindfulness-based interventions (MBIs) in common psychiatric disorders (Hedman-Lagerlöf et al., 2018). Specifically, MBIs that emphasize interoceptive skills have been shown to have beneficial transtherapeutic effects in both mental and general health (Greeson et al., 2014; Farb et al., 2015; Gibney et al., unpublished).

Most authors agree that mindfulness involves a purposeful awareness of one’s experience, including interoceptive experience, unfolding in the present moment, free from reactivity and identification with the experience. Accordingly, mindfulness methods are being increasingly used in clinical interventions on the basis that they assist emotion regulation (Hölzel et al., 2011a; Tang et al., 2015). For example, mindfulness of breath has been shown to produce a generalized reduction in amygdala response to emotional stimuli that is maintained during non-meditative states (Desbordes et al., 2012). Several authors have proposed that emotion regulation effects produced by mindfulness training are largely due to training in executive control (e.g., Bishop et al., 2004; Teper et al., 2013) and to the dynamic interaction between cognitive and interoceptive processes (Cayoun, 2011; Hölzel et al., 2011b). Mindfulness training has also been shown to produce analgesic effects in chronic pain patients (Kabat-Zinn et al., 1985; Zeidan and Vago, 2016; Cayoun et al., 2017) and with acute experimental pain (Shires et al., 2020).

However, as also noted by others (e.g., Gibson, 2019), under the umbrella of ‘mindfulness interventions,’ the methods that patients are asked to practice can differ greatly because the original instructions from traditional Buddhist teachings, which are already diverse, have been modified to varying degrees to adapt to various mental health conditions and clinical populations (e.g., Linehan, 1993; Kristeller and Hallett, 1999; Segal et al., 2002; Bowen et al., 2009). Some of these adaptations have prioritized certain mindfulness skills (e.g., exteroceptive awareness) and deemphasized others (e.g., interoceptive awareness and equanimity) to increase congruence with the targeted symptomatology. It is not clear whether such diagnostic-specific adaptations affect therapy outcomes given the central role of interoception in a wide range of mental health disorders. While it is well established that core mindfulness principles can be used for a wide range of symptoms and conditions (Keng et al., 2011; Greeson et al., 2014; Cayoun et al., 2019), the active mechanisms that permit a transdiagnostic benefit are not well translated into clinical applications (Grabovac et al., 2011).

Accordingly, we first operationalize mindfulness in behavioral terms and review some of the key findings suggesting that mindfulness practice enhances emotion regulation. We then integrate these observations through the co-emergence model of reinforcement (CMR), which is a neurophenomenological account of operant conditioning initially proposed by Cayoun (2011). We describe how the model provides a more accurate and integrated account of behavior reinforcement, which also explains how the mechanisms of action in mindfulness help decrease the typical symptoms of emotional disorders. We will then discuss the involvement of interoception in the encoding, storage and retrieval of emotional memory and the benefits that mindfulness meditation can provide in reprocessing autobiographical memory through interoceptive awareness and equanimity. We reconceptualize emotional disorders as an over-identification with, and inability to extinguish, the interoceptive memory trace associated with the disorder. We then describe how specific mindfulness practices can be used to overcome this deficit transdiagnostically.

## Operationalization of Mindfulness

Mindfulness is the usual translation of the Pali word *sati*, which is suggested to mean ‘remembering with wisdom,’ *sati-nepakkena* in Pali (Levman, 2017). As traditionally taught in the Theravada tradition of Buddhist psychology, mindfulness is a means to an end, cultivating wisdom and reducing suffering, rather than a goal in itself (Gethin, 2011). It is understood as a mental skillset or ‘tool of investigation’ to be developed in order to decrease suffering by cultivating one’s insight into the impermanence of all phenomena, including the sense of self. This is done in part by decreasing one’s identification with one’s sense of self, which is encountered subjectively through meditation in the form of thoughts and body sensations arising and passing in conscious awareness (Hart, 1987; Bodhi, 2000; Grabovac et al., 2011).

During mindfulness of breath meditation, trainees first learn to sustain attention on their breath and learn to recognize thoughts that emerge spontaneously in consciousness, inhibit the tendency to identify with, and react to, these thoughts, and refocus attention on the breath as soon as possible (Brahm, 2006; Nanamoli and Bodhi, 2009). Trainees inhibit the learned response (to engage with the thought) only; they do not inhibit the thought itself. In Western applications of mindfulness, this process has also been termed “decentering” (Williams et al., 2000). This includes inhibiting the aversion that one might experience when being distracted by thoughts while trying to focus on the breath during meditation. Some authors have proposed that this practice requires, besides curiosity and an accepting attitude, the training of at least three higher order executive functions: (1) sustained attention on a target (breath) while holding the task in mind, (2) response inhibition to intrusive stimuli (e.g., thoughts and images) and (3) cognitive flexibility through shifting attention back to the target; the breath (Bishop et al., 2004; Cayoun, 2011). Rather than trying to modify the content of thoughts, trainees develop a degree of inhibitory control over the processes that proliferate thinking, allowing a progressive reduction of intrusive thoughts (Segal et al., 2002; Bishop et al., 2004).

With the widely used Theravada teaching in the Burmese Vipassana tradition of Ledi Sayadaw, U Ba Khin and S. N. Goenka (Ledi, 1965/1999; Hart, 1987), once concentration has been sufficiently developed, the emphasis of effort is on developing interoceptive awareness and equanimity. This requires passing one’s attention (‘scan’) systematically throughout the body, starting with small sections at a time, feeling body sensations, accepting them unconditionally, and moving attention to the adjacent part, to cultivate an increasing ability for interoceptive awareness and acceptance. Meditators also train to inhibit their habit of identifying with body sensations and their habitual responses associated with hedonic tone (pleasant, neutral and unpleasant experiences). This enables a progressive ability to perceive phenomena more objectively – as impermanent and therefore impersonal – and with less reliance on preconceived ideas and emotional reactivity. Since body sensations are considered to be the basic building blocks of emotions (Barrett, 2006), preventing reactivity to body sensations helps regulate emotions (Hart, 1987; Cayoun et al., 2019).

Accordingly, mindfulness meditation practiced in this way, for at least two millennia (Analayo, 2006), has been operationalized in behavioral terms as an exposure to internally generated stimuli (thoughts images and body sensations) that requires a deliberate effort to prevent the conditioned response to both distressing and pleasurable cues, and the development of greater objectivity (less reliance on mental representations) and ‘equanimity’ (Cayoun, 2011; Hölzel et al., 2011b; Farb et al., 2015). Equanimity has been defined as “an even-minded mental state or dispositional tendency toward all experiences or objects, regardless of their affective valence (pleasant, unpleasant or neutral) or source” (Desbordes et al., 2015, p. 357).

## The co-emergence Model of Reinforcement

It has been repeatedly shown that the experience of emotion is determined by interoception (Garfinkel and Critchley, 2012; Damasio and Carvalho, 2013; Seth and Critchley, 2013; Pistoia et al., 2015). Barrett (2006) noted that the interoceptive quality of affect is “a basic, invariant building block of emotional life that can be observed in self-reports of experience as well as in virtually all instrument-based measures of emotion” (p. 50). There is considerable evidence that interoception is associated with activation of various regions of the insular cortex (Craig, 2003), especially the anterior insula (Menon and Uddin, 2010; Garfinkel and Critchley, 2012), although exceptions have been observed (Damasio and Carvalho, 2013; see also Uddin, 2014, for a review). With regards to neural correlates of emotional awareness, there are indications of dynamic interactions between interoceptive cues of emotion and attention in the anterior cingulate cortex (ACC), linking attention and response selection to interoception (Lane et al., 1998). Taken together, these studies suggest that emotion is felt within the framework of the body, with thinking and feeling co-emerging and depending on one another to manufacture behavior, as outlined by the CMR.

The CMR is a unifying approach to behavior maintenance and change that integrates elements of cognitive neuroscience, learning theory, and the ‘five aggregates,’ the essential human information processing framework expressed in the Buddhist psychological system (Narada, 1968; Nyanaponika, 1996; Bodhi, 2000). Since emotional reactivity is both a common life experience and a transdiagnostic process in most psychological disorders (Feldman et al., 2015), its behavioral representation must be as generally applicable as possible. Accordingly, the CMR is necessarily generic to permit transdiagnostic applicability. In line with this purpose, it is presented here as simply as possible, by means of four generic functional components (for more comprehensive descriptions, see Cayoun, 2011 and Cayoun et al., 2019).

### Basic Principles of Equilibrium States

**FIGURE 1:**
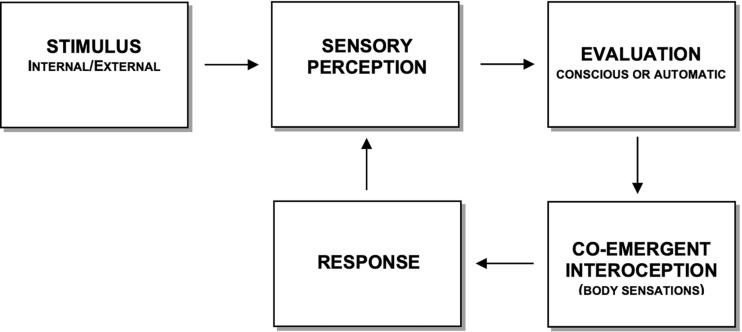
Components of the co-emergence model of reinforcement during equilibrium in information processing (adapted from Cayoun, 2011). The response is a direct consequence of interoceptive experience and is not directly dependent upon the stimulus or cognitive evaluation. The arrow with a dotted line represents a faster route, which serves to process highly automated and instinctive signals (e.g., survival reflexes), where associations between the perception of the stimulus and the interoceptive “meaning” no longer appears to require evaluative processing.

The non-pathological functioning of the model components is presented in Figure 1. The model suggests that stimuli perceived as personally significant are encoded interoceptively before behavioral output is determined, in line with mechanisms of interoceptive salience proposed by Menon and Uddin (2010) and Uddin (2014), as per the following sequence. Once an external or internal stimulus (Stimulus in Figure 1) has been perceived through the senses (Sensory Perception in Figure 1), it is categorized and processed through evaluative filters of self-referential relevance, such as personal values, beliefs and schematic models, autobiographical memories, personality traits, perceived needs, and cultural predispositions (Evaluation in Figure 1). As a rough guide, their expression is often preceded by pronouns such as “I,” “my,” “me,” “ours” (e.g., I am sure people at work think I can’t cope”). These can be implicitly embedded in “you,” “your,” “yours” and “theirs,” when comparisons with self-references are made. This evaluative process may be conscious, especially when processing novel or complex stimuli (e.g., “I realize that I am not very good with statistics”), or more rapid and subconscious/automatic when the need for conscious updates in evaluation is unnecessary.

Evaluations can be placed on a continuum of self-reference, in terms of perceived personal importance. Self-reference includes stimuli associated with one’s sense of self and its associations (e.g., one’s views); it is not limited to one’s immediate or past personal experiences. Toward one end of the continuum, evaluations are increasingly descriptive in nature, neutral, and stimuli appear as least personally important. Toward the other end, evaluations are self-referential in nature and contain likes and dislikes associated with personal judgment and affect. This is congruent with Teasdale and Barnard (1993) multi-level interactive cognitive subsystems model of cognitive architecture, where a ‘central engine’ processes ‘propositional’ (factual) and ‘implicational’ (self-referential) thoughts.

Central to the CMR is that self-referential evaluations ‘co-emerge’ simultaneously with body sensations (Co-emergent Interoception in Figure 1), whereby the more a stimulus is evaluated as being personally important, the more intense the co-emerging body sensation. The term ‘co-emergence’ refers to the experience that thinking and feeling are occurring simultaneously, even though there may be a short and often indistinguishable firing delay between the two neural substrates that subserve these functions. Note that interoceptive experiences that precede Evaluation (e.g., sudden pain or cold sensation due to cold weather) are non-co-emergent internal stimuli, as they pertain to the Stimulus component. Moreover, the hedonic tone of co-emerging interoception (valence) is determined by the agreeableness of the evaluation. The more agreeable an evaluation, the more pleasant the co-emerging interoceptive experience is likely to be. Likewise, least agreeable evaluations co-emerge with the most unpleasant body sensations. The findings of Barrett and other emotion theorists also share the view that ‘core valence,’ in terms of the interoceptive hedonic tone of mood, is an invariant property of emotional life that integrates an evaluation system (Russell, 2003; Barrett et al., 2004; Barrett, 2006). They propose that interoceptive valence derives from the psychological process of ‘valuation,’ defined as “a simple form of meaning analysis in which something is judged as helpful or harmful in a given instance, producing some change in core affective state [and] judgments about whether stimuli or events are helpful or harmful, rewarding or threatening, help to influence the valence property of core affect” (Barrett, 2006, p. 39). Self-referential evaluations and their co-emerging interoception are stored and subsequently retrieved in a co-emergent way, as will be discussed later.

The fourth function of the CMR is the response (Reaction in Figure 1), which is dependent upon the intensity and quality (hedonic tone) of body sensations. The proposition that self-referential evaluation and interoception combine to guide behavior is supported by several studies of the neurobiological bases of emotion (e.g., Lane et al., 1998; Gerber et al., 2008). Note that a reaction can be constrained in cognition, as in obsessive and ruminative thinking, or making a rational decision. Unless trained to respond otherwise with methods that promote equanimity, reactivity is a function of interoceptive intensity. The more a body sensation is intense, the more likely it is that a covert or overt reaction will occur, irrespective of whether the sensation is pleasant or unpleasant. With regards to the type of reaction, the CMR proposes that it is a function of hedonic tone. The more pleasant a body sensation, the more likely it is to lead to attachment and a desire for more. Unpleasant body sensations tend to lead to any form of experiential avoidance. Reinforcement occurs once the response serves to either decrease unpleasant sensations (negative reinforcement) or increase pleasant ones (positive reinforcement). Once a response has occurred, it feeds back into Sensory Perception and does not require exteroceptive information to maintain continuity of information processing. This closed loop formation is easily noticeable during maladaptive cognitive and metacognitive processing, such as ruminative thinking and worry about worry, typical of generalized anxiety disorder. As suggested by Barrett (2006, p. 40), “the intensity of a core affective experience results in a perceived urgency to act,” an observation supported by several studies (e.g., Barrett, 2004; Barrett et al., 2004; Barrett and Niedenthal, 2004).

In standard operant conditioning (Skinner, 1953), learning takes place when the behavior ‘operates’ on the environment to generate consequences. Behavior is either increased or decreased by the organism as a function of the resulting consequence of that behavior. If the behavior leads to a desired outcome (a rewarding ‘consequence’), the organism is likely to increase the frequency of the behavior. If the behavior leads to an undesired outcome (a non-rewarding or punishing consequence), the organism is likely to decrease the frequency of the behavior. This approach gives minimal, if any, consideration for the role of internal contexts, such as cognition and interoception. In contrast, the CMR suggests that the consequence on which the next behavior operates is the change in interoceptive feedback, and is not dependent on an external consequence caused by the previous behavior. Hence, the CMR proposes that we do not react directly to external triggers; we react according to the sensation we feel consciously or subconsciously in the body.

Neurologically, this mechanism is likely to be attributable to the salience network, forming important linkages between the anterior insula and the anterior cingulate that function to segregate the most relevant among internal and extra-personal stimuli to guide behavior (Menon and Uddin, 2010). There is recognition that this network is of fundamental importance for mental life (Medford and Critchley, 2010). In particular, interoceptive signals produced in the dorsal anterior insular cortex play a key role in assigning significance to stimuli in daily experiences (Damasio, 1999; Critchley et al., 2004; Uddin, 2014; Goodkind et al., 2015). There is also some evidence of ongoing synchronized functionality between the default mode network and salience networks (Doll et al., 2015), which may be reflected in the CMR by the continual interaction between Evaluation and Co-emerging Interoception. The notion that learned responses rely on interoceptive experience, even when the stimulus is external to the person, differentiates this approach from the conventional understanding of behavior reinforcement. It follows that these mechanisms apply to other types of conditioning modalities, such as observational learning and classical conditioning. As such, all learning principles rely on the co-emergence of cognition and interoception. This alone has significant implications for clinical interventions where exposure therapy is recommended.

### System in Disequilibrium and Psychopathology

**FIGURE 2:**
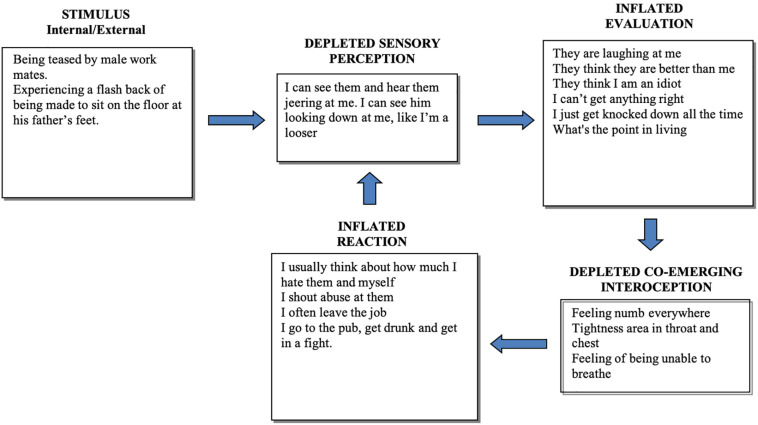
Representation of a disequilibrium state for a young man with a history of trauma, criminal history, drug and alcohol problems and self-harm. Sensory perception of the situation is depleted and therefore contaminated by evaluative and self-referential cognitions. Note that his behavior (Inflated Reaction) is reinforced because it reduces his numbness and other interoceptive discomfort.

**FIGURE 3:**
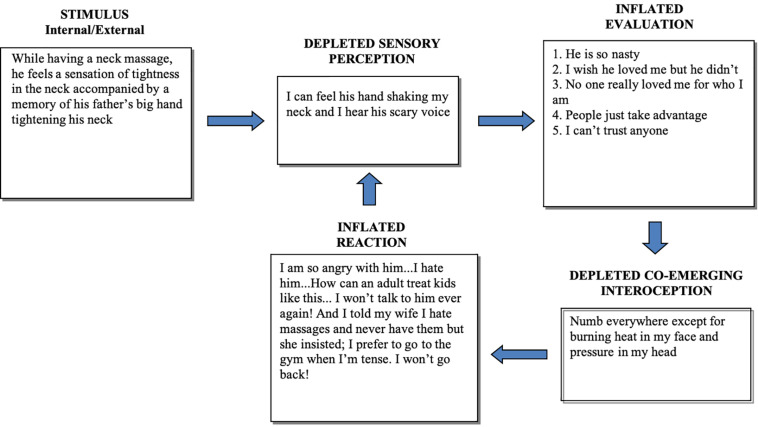
Representation of a disequilibrium state in a middle-aged man with a history of childhood physical abuse and dysregulated mood. In this case too, sensory perception of the situation is depleted and contaminated by intrusive fearful memories, leading to self-referential cognitions. His reaction (avoidance of sensory cues) is reinforced by the prevention of dissociative and other interoceptive discomfort.

The four processing components are in a state of disequilibrium during a stressful experience. ‘Disequilibrium’ in this context means that the balance between components necessary for optimum cognitive and emotional functioning is disrupted by the additional processing demand of some components at the cost of processing capacity of others. When a person is distressed, attention resources are depleted from sensory processes (Sensory Perception and Co-emergent Interoception) and reallocated to Evaluation and Reaction, creating a functional disequilibrium between components. This is pictorially represented through the case examples in Figures 2, 3 by larger boxes for expanded processing and smaller boxes for depleted processing. These are examples of a disequilibrium state for patients with a complex post-traumatic stress disorder (PTSD). Figure 2 shows the CMR description of a 23-year-old male, manual laborer with complex PTSD and substance use disorder. Figure 3 shows the CMR description of a 38-year-old male, unemployed with complex PTSD and persistent depressive disorder.

Inflated reactivity prevents full access to sensory perception, as exemplified by dissociative states during experiences of anxiety. This is supported by findings that basic sensory ability correlates negatively with stress (Posserud et al., 2004) and both high-arousal negative emotions (e.g., fear and anxiety) and pleasure through appetitive arousal (e.g., food and sexual reward) have a narrowing effect on attentional scope and conceptual attention is also reduced by high attachment states (Friedman and Forster, 2010). In other words, whether intense interoceptive cues are associated with aversion or craving, the scope of attentional capacity narrows. This corresponds to depleted Sensory Perception in Figures 2, 3. In contrast, positive emotional states that do not involve craving (e.g., a sense of happiness) have an expansive effect on attentional scope (Friedman and Forster, 2010). This corresponds to the system in equilibrium state, where the Sensory Perception component functions at full capacity (Figure 1). This lack of perceptual effectiveness limits the accuracy of perceptual ability and leads to an overreliance on assumptions (Evaluation) held in memory, such as cognitive schemas.

There are also indications that at a biological level, reinforcement depends on the ability of the nucleus accumbens (NAc) to predict an outcome (O’Doherty et al., 2004). Indeed, both conditioned reward and conditioned aversion are associated with the activation of the NAc, which can encode differentially the hedonic value of the same stimulus based on learned associations (Roitman et al., 2010). As such, an important aspect of reinforcement is the influence of context, which can be nicely illustrated by the following case example of a person experiencing radically different hedonic values of the same stimulus (pain) based on learned associations. Consider two different situations in which the same person encounters the same experience of physical pain, one due to domestic violence and the other to masochistic sexual behavior. In both situations, a disequilibrium state is expected. The nervous system depletes attention from the more objective sensory appraisal networks and reallocates attention to the *learned meaning* of the stimulus, which leads to radically different co-emerging body sensations, and presumably opposite reactions. When the person experiences physical pain (Stimulus) caused by domestic violence, the unpleasant body sensations that co-emerge with the negative evaluation of what is happening leads to an aversive response in an attempt to decrease the duration and frequency of unpleasant co-emerging body sensations. In contrast, when the person experiences the *same* physical pain (Stimulus), but caused by masochistic sex, he or she will interpret it as pleasant because of its association with a safe and agreeable context. The pleasant body sensations that co-emerge with the positive evaluation then leads to a craving response in an attempt to increase the duration and frequency of co-emerging pleasant hedonic tone; the ‘reward.’ The significance of context in conditioning principles has been highlighted by others (e.g., Hayes et al., 2002).

A non-pathological disequilibrium state reflects only a transient reallocation of resources that may serve a survival purpose by allowing the rapid evaluation of potential threats and immediate deployment of defensive reactivity, in line with the function of the salience network (Menon and Uddin, 2010; Uddin, 2014). For instance, when a stimulus is ambiguous but could be threatening, rapid retrieval and use of mental representations of the stimulus replaces the actual sensory perception of the stimulus. In other words, “what it is” is replaced with “what it is like,” and often “what it is like *for me*,” producing spontaneous co-emerging interoception of which the individual may not be aware. He or she then reacts, immediately assuming that the internal experience is an accurate measure of what the stimulus represents. This is proposed to be invariant across situations and is not limited to ambiguities or threats. The model suggests that the same principle applies to pleasant situations, such as infatuation and appetitive behavior.

In pathological states of disequilibrium, the model posits that the increased processing of self-referential cognition and reactivity, and the reduced capacity to process sensory signals, is learned and sustained beyond the reasonable need for reactivity. The individual identifies this state as their own. Some of the cognitive and behavioral correlates of pathological disequilibrium include dissociative states (markedly depleted Sensory Perception and co-emergent Interoception), ruminative and catastrophic thinking and self-preoccupation, accompanied by over-reactivity, such as impulsivity and avoidance (markedly inflated Evaluation and Reaction). What defines a pathological state of disequilibrium is both the extent or ‘severity’ of the disequilibrium, and its persistence over time, reflected in the degree of impaired functioning in daily life. In particular, a lack of interoceptive awareness and equanimity contributes to relapse into emotional disorders and chronicity, which in turn contributes to the maintenance or worsening of dysfunctional personality traits. A sustained disequilibrium state is thought to lead to self-identification with this state, habituation, and eventually a sense of normality in being over-judgmental and over-reactive, and less aware of interoceptive and other sensory cues.

One of the clinical predictions of this model is that the more established and pronounced the disequilibrium state, the more likely it is to initiate or maintain psychopathology. This mechanism is supported by the growing multidisciplinary findings of depleted interoception and inflated self-referential cognition (e.g., rumination) in mental health disorders (Farb et al., 2010; Khalsa et al., 2018). For instance, studies show a consistent pattern of impaired interoception in depression and associated somatic comorbidities, and indicates that the core symptoms of depression may be caused by disrupted interoceptive-exteroceptive integration (Harshaw, 2015). People with major depressive disorder show bilateral decreased activation in the dorsolateral insula during an interoceptive attention task (Avery et al., 2014). There are also indications that high levels of ruminative brooding in depressive and anxious states (overinflated Evaluation and Reaction in Figures 2, 3) are associated with decreased interoceptive awareness (i.e., depleted Sensory Perception and Co-emergent Interoception in Figures 2, 3) (Lackner and Fresco, 2016). Moreover, two studies investigated the interoceptive capacity of individuals with differing degrees of suicidality (Forrest et al., 2015). The authors found an impaired interoceptive capacity in individuals with suicidality compared to controls, and access to interoception decreased as a function of symptom severity. These studies are consistent with the accumulating evidence of abnormal functioning of specific subdivisions of the insula within the salience network, which is a feature of numerous neuropsychiatric disorders (Uddin, 2014; Goodkind et al., 2015; Peters et al., 2016), as suggested by the CMR in chronic disequilibrium state.

### Recreating Equilibrium

Another central tenet of the CMR is that as disequilibrium can be learned, it can also be unlearned. Restoration of a more balanced state between components assists in the extinguishment of unhelpful behavior, helps prevent relapse through minimizing reactivity to interoceptive cues, and reduces the underlying neural activation associated with disequilibrium states. Recent psychophysiological studies using inhibition tasks show that increasing interoceptive awareness improved both unintentional and intentional response inhibition (Rae et al., 2018, 2019). During mindfulness meditation, by focusing on (‘scanning’) body sensations (increased interoceptive processing) while remaining equanimous (decreased self-referential judgments and reactivity) during regular mindfulness meditation, one can recreate equilibrium between imbalanced components. Support for this proposition comes from studies showing that emotional reactivity is associated with increased self-referent evaluations and the suppression of viscerosomatic networks (disequilibrium state), whereas the practice of mindfulness activates networks that help balance the regulatory responses to emotions, while allowing non-sensory visceral information to be experienced (equilibrium state) (Farb et al., 2007, 2010; Avery et al., 2014; Tomasino and Fabbro, 2016). Experienced mindfulness meditators show enhanced activation of interoceptive representations in the insular and somatosensory cortices (Lazar et al., 2005; Hölzel et al., 2008).

Accordingly, we suggest that mindfulness meditation teaches ideal skills for the purpose of promoting equilibrium between components of information processing reflected in the CMR. We also suggest that the CMR can be both a useful rationale for using mindfulness training in clinical practice and a convenient means of operationalizing the active mechanisms of mindfulness training. This is supported by an investigation of mindfulness mechanisms comparing non-meditators (*n* = 284) with meditators (*n* = 386) trained in a variety of mindfulness methods and traditions, which showed greater body awareness and non-reactivity in meditators (Cebolla et al., 2017). A recent study also examined the effects of three most commonly used meditation methods used during mindfulness interventions (mindfulness of breath, body scanning and loving-kindness meditations) on the levels of mindfulness, concentration, self-compassion, emotional regulation and experience, and life satisfaction (Kropp and Sedlmeier, 2019). The authors found that body scanning meditation showed greater effects on almost all dependent variables than those in the other meditation conditions. These studies support our observations that interoceptive awareness and inhibitory control are key mechanisms in mindfulness practice. This point was recently emphasized by Gibson (2019), whose review shows that it may be more accurate to link many of the identified benefits of mindfulness practice to increased interoception and its neurological correlates.

## Proposed Roles of co-emergence in Memory Processes

Since conditioning principles are heavily reliant on memory, we now discuss the roles of co-emergence principles in memory to provide a broader integration of co-emergent information processing. In particular, the CMR puts forward several propositions that have implications for clinical interventions, especially where autobiographical memories contribute to symptom proliferation.

### Co-emergent Encoding and Retrieval

The first postulate is that experiences that involve self-referential (including schema-based) processing are encoded in memory, and later retrieved, co-emergently, i.e., with concomitant conscious or non-conscious body sensations. This is easily observed in people with PTSD, where intrusive traumatic memories co-emerge with viscerosomatic sensations, creating a distressing sense of ‘nowness’ (Nader, 2015; Payne et al., 2015). Whether a person’s mood can serve as a distinctive interoceptive context for learning and retrieval of memory is not a novel question in cognitive science. Mood-dependent memory research emerged over 40 years ago through the work of Bower et al. (1978), and has shown that the emotional valence associated with a memory target at the encoding stage can serve as a retrieval cue. Accordingly, Bower et al. (1981) proposed that an emotion is a unit within a semantic network that encodes memories.

Recent fMRI studies confirmed this observation. Xu et al. (2014) found that the practice of non-directive meditation triggers spontaneous episodic memory and emotional processing. Pais-Vieira et al. (2016) explored how attention oriented to interoception and exteroception during emotional experience can differentially influence memory encoding and recall. They found evidence that paying attention to interoceptive cues of emotion promotes the formation of emotional memory through the activation of brain networks associated with interoceptive awareness of emotion, such as anterior insula and dorsal ACC. This is consistent with Menon and Uddin (2010) salience network, which may be part of the neural structures that subserve the dynamics of co-emergence. Pais-Vieira et al. (2016) results demonstrate that interoceptive attention promotes the retrieval of emotional memory.

Additionally, the CMR implies that the co-emergence of self-referential evaluations with interoceptive cues subsequently help recognize personally important stimuli during the course of a lifetime. This is proposed to be a fundamental principle for the formation and perpetuation of schemas; the maintenance of unhelpful schemas relies on reactivity to body sensations. We reason that such reactivity can persist when the chosen therapy does not take interoception sufficiently into account, increasing the probability of relapse. Since one reacts to, or because of, body sensations (Hart, 1987), not being sufficiently aware of them leads to schema-based evaluations and automatic reactivity, as exemplified above in Figures 2, 3.

This conceptualization implies that since all significant life events, including schema-related and trauma memories, are encoded and stored with co-emergent body sensations, scanning the body with equanimity during mindfulness meditation is also ‘scanning through our past’; our ‘dormant’ autobiographical memories which can be reactivated through the interoceptive stimulation triggered by ‘scanning’ the body. Allowing the full emergence of interoceptive experience during body scanning leads to experiencing sensations that are sufficiently similar to those experienced in the past, capable of acting as memory cues for past emotions—many of which operate below the level of conscious awareness. Based on the CMR, remaining aware of body sensations and equanimous, while not identifying with the experience and observing it for what it is, maintains an equilibrium state and extinguishes the reactive response. We propose that cognitive schemas are experienced co-emergently and that some of the reported benefits of practicing body-scanning methods during mindfulness meditation are attributable to the desensitization of such schemas through interoceptive exposure.

### Recall Facilitation Through Interoception

The second postulate is that the probability of retrieval in autobiographical memory is proportional to the intensity of interoception that co-emerged at the encoding stage; although the amount of memory reconsolidation over time will also affect decay (Tronson et al., 2006). Nader (2015) noted that the *significance* of a memory facilitates remembrance, as understood from over five decades of memory research. For instance, early studies which examined people’s memory for materials that evoked emotional reactions of differing intensities showed that memory retrieval of an event increases with the emotional intensity experienced at the time of that event (Kanungo and Dutta, 1966; Dutta and Kanungo, 1975). The specific relationship between interoceptive awareness and processing and emotional memory was more recently demonstrated through psychophysiological studies (e.g., Pollatos et al., 2005, 2007; Pollatos and Schandry, 2007), which provide support for the notion that memory retrieval is a function of interoceptive intensity.

Even in severe traumatic conditions where patients are not always capable of accurately or fully recalling obvious features of the traumatic experience, poor recall is likely to be associated with poor encoding. Experiencing a dissociative state during the traumatic event prevents the co-emerging interoceptive experience that would usually accompany such an event. The CMR explains this in terms of disequilibrium state, whereby attention in both Sensory Perception and Co-emerging Interoception components is most severely depleted, limiting one’s ability to fully feel the emotional content of the experience. The experience of dissociation, such as numbness, during traumatic events is well documented (e.g., van IJizendoorn and Schuengel, 1996; Dutra et al., 2009). In turn, reduced interoception causes poor memory retrieval, as noted by Immordino-Yang and Damasio (2007).

### Co-emergent Encoding Prevents Decay

The third postulate is that memory retention is a function of co-emergence mechanisms and is aligned to evolution and survival needs. While propositional (non-self-referential) memories, such as the brand of an ordinary car that belongs to someone else, are not usually perceived as important to remember, remembering to pick-up the children from school is. In the former case, the memory of the unimportant stimulus is not encoded with sufficiently intense co-emerging body sensations, making it more prone to decay. In contrast, in the latter case, provided our children are important to us (‘*my* children,’ part of *me*), the encoding includes co-emergent interoceptive cues and is therefore thought to activate insular and subcortical networks. The evidence that memory consolidation relies on interoceptive activation in the insular cortex was provided by several studies (Bermudez-Rattoni et al., 2005; Contreras et al., 2012; Bermudez-Rattoni, 2014).

Accordingly, we are proposing that the depth of neural network activation decreases the likelihood of forgetting information that was deemed important at the encoding stage of memory. This includes all stimuli associated with survival needs, whether in the form of threats or means of reproduction. More neutral memories do not need to rely on emotional (interoceptive) cues to prevent decay and require mnemonic strategies to survive, such as rehearsal. It is well demonstrated that emotional memories trigger mechanisms that modulate consolidation, whereas neutral memories fail to do so (McGaugh, 2004; Nader, 2015).

We hypothesize that one of the main purposes served by co-emergent encoding is to assist survival by decreasing the probability of memory decay and permitting successful memory retrieval of what is perceived as personally important (e.g., threat recognition and other survival needs). Updates on whether a memory that should remain personally important can be made as often as necessary, either through reconsolidation processes or through preventing them (Nader et al., 2000; Nader and Hardt, 2009). This allows each individual to maintain or ‘outgrow’ and relinquish unnecessary emotional memories, such as those of childhood trauma. During mindfulness meditation, if the practitioner is accurately trained to develop equanimity while feeling body sensations, potential spontaneous recall of painful memories will not only appear acceptable, they will also provide a context for desensitization and eventually neutralization of learned reactivity to distress cues (Kerr et al., 2013; Cayoun, 2015; Hölzel et al., 2016). Clinical interventions that use interoceptive exposure to desensitize traumatic memories show tangible benefits (Leitch, 2007; Parker et al., 2008; Payne et al., 2015) and improved functional connectivity between the salience network, the default mode network and the ACC (Boyd et al., 2017).

## Co-Emergence Model Integration Within Therapy

### General Implications for Therapy

We propose that the unlearning process of an established disequilibrium state is inherent in mindfulness meditation and is a ‘transtherapeutic’ mechanism (Greeson et al., 2014) that can be embedded in most clinical interventions, including crisis intervention and chronic pain treatment (e.g., Cayoun et al., 2017; Mozafari-Motlagh et al., 2019). As put forward by the CMR, since reactivity is reinforced by its consequence on interoception (Cayoun, 2011), interoceptive awareness and inhibitory control promote interoceptive desensitization and behavior change. This was clearly demonstrated by behavioral studies pioneering interoceptive desensitization in panic disorder, and the numerous studies which have since been carried out across a range of anxiety disorders (see Boettcher et al., 2016, for a review). These studies led to the conclusion that interoceptive exposure can be used as a transdiagnostic intervention (Boswell et al., 2013) and constitutes an important active mechanism in Eustis et al.’s (2020) Unified Protocol for anxiety disorder. However, Boettcher and Barlow (2019) also found that interoceptive exposure implemented on its own for claustrophobia was less effective than the same for panic disorder, and that situational exposure was also required to decrease claustrophobia symptoms. Their results showed that interoceptive exposure on its own was most effective when the feared trigger was generated internally, whereas externally generated fear triggers, such as social cues, also require exposure to external situations. While the idiosyncrasies of a disorder guide the direction for the contextual applications of the model (e.g., exposure to perceived dirt in the case of obsessive-compulsive disorder), the fundamental principle remains the same for emotional disorders: the central mechanism of distress is a disequilibrium state. If a person’s psychological disorder is case-conceptualized in terms of a more or less established disequilibrium state, a congruent intervention is one that attempts to re-establish equilibrium, irrespective of the disorder.

There are two important clinical implications of this conceptualization. One is that all mental health disorders benefit from interoceptive exposure, where patients are exposed to their body sensations while not identifying with them and prevent their learned reactions. The other is that learning to notice the incessant flow of co-emergence of ‘mind and body,’ including how an unhelpful thought co-emerges with an unpleasant body-sensation, promotes well-being and the prevention of relapse. The CMR posits that since ‘relapsing’ involves the reactivation of a past disequilibrium state from memory, it ‘reconsolidates’ the disorder’s memory trace. Hence, we reconceptualize recurrent relapses as *a consequence of the inability to extinguish the co-emergent memory trace of the disorder* (i.e., how unpleasant or numb it feels in the body when one thinks about the world in a certain way, and how one usually reacts in order to feel relieved). This also implies ‘living in the past.’ Hence, the CMR is a viable multidisciplinary rationale for the integration of mindfulness skills, such as interoceptive desensitization, in standard therapies.

Another important implication is the reconceptualization of the focus of therapy. A hypothesis implied in the CMR is that the increased generalization and worsening of unhelpful behavior, such as avoidance or hypervigilance, is a function of a strengthening disequilibrium state. It follows that, with time, such reinforced disequilibrium state will affect other areas of life functioning. This is often shared by patients with generalized anxiety disorder, who explain that their anxiety started with mild OCD or trauma symptoms earlier in their life and eventually generalized to multiple contexts over the years. When the disequilibrium state has become pervasive across time and context, the psychopathology is either considered to be ‘chronic’ or the expression of a disordered personality. Conditions such as borderline personality disorder, for example, are good reflections of a pervasive disequilibrium state. In this case, it may be better called a *disequilibrium trait* because the person over-identifies with being inflexibly over-judgmental and over-reactive, perceiving disequilibrium as normality.

Nonetheless, in contrast with conventional belief that personality factors remain consistent across time and context, we suggest that they can change through targeting their maintaining factor, hypothesized in the CMR as the subconscious continual reinforcement of disequilibrium state and its neurobiological substrate. This view is supported by studies of mindfulness methods that emphasize interoceptive exposure and non-identification with body sensations as the core of the practice, which is a fundamental skillsets traditionally taught in mindfulness meditation, known as ‘contemplation of feeling’ (Hart, 1987; Walshe, 2012)—note that ‘feeling’ here refers to interoception. Change in personality factors was observed in a recent study of violent male offenders (*N* = 403), showing that mindfulness, alexithymia, and empathy significantly explained an incremental amount of variance in traits of antisocial personality disorder in separate regression analyses (Velotti et al., 2019).

### Reappraisal Facilitation

The ability to reappraise emotional reactivity during aversive events is an important aspect of both crisis intervention and relapse prevention, because it is central to healthy emotion regulation. However, reappraisal attempts alone seem to be minimally effective in decreasing the activation of limbic regions and associated emotional reactivity (Craig, 2002). From a co-emergence point of view, this may be partly due to the reduced ability to perceive stimuli accurately during a stressful experience (e.g., Friedman and Forster, 2010; Kang et al., 2013), as represented in the model by depleted Sensory Perception in a disequilibrium state (Figures 2, 3). Poor access to accurate perception leads to misinterpretations (inflated Evaluation in Figures 2, 3). Importantly, poor ability to reappraise during distress may also be due to a lack of interoceptive awareness. We suggest that body sensations ‘hold’ unhelpful thoughts via co-emergence. Consequently, the coupling of unhelpful thoughts and interoceptive experience is difficult to overcome when standard cognitive reappraisal (Ellis, 1962; Beck, 1967) is not accompanied by interoceptive exposure and acceptance. This may better explain the difficulty in halting ruminative thinking in anxiety and depression or intrusive obsessive thoughts in OCD, for example. Unless the patient is able to focus on the co-emerging body sensation with a degree of equanimity, these dysfunctional thoughts are likely to persist. In support of this view, the results of Füstös et al. (2013) showed that interoceptive awareness facilitates cognitive reappraisal, which was accompanied by reduced arousal. Although mindfulness meditation does not include the typical style of cognitive reappraisal used in cognitive therapy (Beck, 1967), it requires a clear discernment of the mind-body interaction during emotional distress and ‘holding in mind’ the notion that ‘there is no need to react because it will pass.’ At least in the early stage of learning the practice, the meditator reappraises the situation in these terms and prevents the maintenance and proliferation of unhelpful thoughts during the meditative experience.

### Benefits of CMR Integration

It has been argued that the currently assumed causality of relationship between diagnosis and therapy for conditions, such as major depressive disorder, lacks scientific evidence (Stojanov et al., 2011). The authors stress the lack of relationship between therapeutic regimens and suspected pathogenesis, and the inadequacy of tools to diagnose and delineate a psychological disorder, among other obstacles to an evidence-based psychiatry (see Stojanov et al., 2011, for comprehensive discussion). In relation to these observations, there are several advantages of having the CMR as a theoretical underpinning for clinical intervention: the standard classification of emotional disorders matters less to the clinician since the CMR improves homogeneity of categories (a continuum of disequilibrium state); therapy can be directed to core symptoms (unawareness of co-emergence dynamics and interoceptive conditioning); and markers and inquiry do inform core symptoms (since the locus of reinforcement is interoception, the target of exposure is necessarily the interoceptive experience occurring during distressing events). For example, standard exposure to social cues for people with social anxiety disorder without prior or concurrent interoceptive exposure tends to yield poor results (Mansell et al., 2003). A recent randomized controlled trial (RCT) tested the generic assumption of the CMR—that recreating an equilibrium state decreases psychopathology—by comparing the efficacy of therapy methods that emphasize cognitive skills with others that emphasize interoceptive and acceptance skills to reduce OCD symptoms (Derakhtkar et al., unpublished). These were Cognitive-Behavior Therapy (CBT), Metacognitive Therapy (MCT), Acceptance Commitment Therapy (ACT), and Mindfulness-integrated CBT (MiCBT). The results showed that all four intervention decreased OCD symptoms in the short-term relative to controls. However, only participants in the MiCBT and ACT groups maintained their gains at follow-up. No significant difference were found between MCT and CBT in the long-term. Whereas CBT and MCT centered their intervention on cognitive and metacognitive processing (respectively), ACT and MiCBT emphasized mindfulness skills, including interoceptive desensitization.

There is also increasing evidence that including interoceptive desensitization to reduce the pathological effects of disequilibrium state has transdiagnostic benefits. Several RCTs have shown that MiCBT, which attempts to re-establish equilibrium state transdiagnostically, is efficacious in treating anxiety and depression in pregnant women (Yazdanimehr et al., 2016) and in women with multiple sclerosis (Bahrani et al., 2017), reducing perceived pain and increasing pain self-efficacy in patients with breast cancer (Mozafari-Motlagh et al., 2019), reducing anxiety and fatigue while improving sleep quality in patients with Multiple Sclerosis (Pouyanfard et al., 2019), and reducing sports-anxiety and pessimism, and increasing flow and adherence in competitive athletes (Scott-Hamilton and Schutte, 2016; Scott-Hamilton et al., 2016). Other studies successfully included specific MiCBT skills that promote the equilibrium state in other mindfulness and acceptance-based methods (e.g., Swain et al., 2013; Senderey, 2017). In a recent RCT, Francis et al. (unpublished) examined the transdiagnostic effects of re-establishing an equilibrium state through an 8-week MiCBT program delivered in heterogenous groups of patients with multimorbidity attending a psychology clinic. The results showed significantly greater decrease in psychological distress in the MiCBT group than in the treatment-as-usual group from pre- to post-treatment and at 6-month follow-up across all disorders included in the sample (*N* = 118). Mediation analysis indicated that equanimity toward body sensations was the most influential mediator of the primary outcome. This study demonstrates the transdiagnostic efficacy of MiCBT and supports its applications with heterogenous groups in community-based primary mental health care.

Moreover, the transdiagnostic applicability of interoceptive desensitization is not limited to its integration in CBT. Any evidence-based approach in which interoceptive desensitization is carefully implemented is likely to promote an equilibrium state, which in turn can be learned if maintained over time through training. For example, Cayoun et al. (2017) tested the CMR in 15 patients with chronic pain and multimorbidity. We hypothesized that creating an ongoing equilibrium state through interoceptive desensitization could assist patients to unlearn the central sensitization effect of chronic pain. Participants were trained in a self-guided 30-s mindfulness-based interoceptive exposure task (MIET), which they were asked to apply to pain sensations each time pain intensity increased during the day, repeatedly over 15 days. Participants were asked to focus attention at the center of the most intense body sensation as objectively as possible and prevent experiential avoidance. Explained through the CMR, they learned not to identify with pain sensations (decreased self-referential evaluation of the stimulus and co-emerging body sensations) and focused on four subcomponents of interoceptive experience (mass, motion, temperature, and cohesiveness) (increased sensory perception) while remaining equanimous (decreased reactivity). The results showed a large and significant reduction in pain anxiety (*p* = 0.001; *d* = 0.96), pain duration (*p* = 0.01; *d* = 0.86), and pain intensity after each 30-s exposure (*p* < 0.001; *d* = 1.37). These effects were maintained, and some further improved, at 2-month follow-up. This may be explained through brain correlates, as the interoceptive network is closely connected to both the nociceptive and affective networks (Khalsa et al., 2018). In a subsequent RCT in experimental pain comparing the MIET to distraction (Shires et al., 2019), the results showed that participants in the MIET group were significantly more able to tolerate induced pain than those in the distraction and control groups (*p* < 0.005, *d* = 1.42 and *p* < 0.005, *d* = 1.06, respectively). Additionally, the degree to which pain increased over the time course of the task was also significantly more reduced in the MIET group, despite higher initial pain levels. These results demonstrated that effort to recreate an equilibrium state in the CMR, by disengaging the habitual self-referential evaluative and reactive components while engaging the sensory/interoceptive components, was sufficient to decrease pain perception significantly more than distraction, which is often used in standard CBT for coping with pain. The Cayoun et al. (2017) MIET study also found that a significant decrease in depression, anxiety and stress occurred after 2 weeks of MIET implementation and even more so at 2-month follow-up (*p* < 0.001; *d* = 0.81). We proposed that the MIET’s requirement for continual use of interoceptive exposure produced increased connectivity in insular functions and top-down inhibition of ascending nociceptive signals and emotional reactivity. We hypothesize that this method produced an immediate and lasting pain reduction by creating an equilibrium state in the information processing system, and that this can be equally achieved with emotional disorders.

## Conclusion and Future Directions

The CMR presents several testable hypotheses related to both behavior reinforcement and memory processes:

• Stimuli that are consciously or subconsciously evaluated as being personally important co-emerge spontaneously with body sensations, the role of which is twofold: evaluate the significance of a stimulus to select an appropriate response, and strengthen memory encoding and storage to improve recall. This process appears to be neurologically subserved by the salience network.
• The intensity of co-emerging body sensations is a function of self-referential evaluation (e.g., the extent to which a situation appears personally important).
• The hedonic tone (pleasant, unpleasant or neutral) of co-emerging body sensations is a function of evaluative agreeableness (i.e., agreeable evaluation of a stimulus co-emerges with pleasurable body sensations and disagreeable evaluation co-emerges with unpleasant body sensations).
• Reactive behavior is a consequence of interoceptive salience and correlates positively with interoceptive intensity (i.e., the likelihood of an emotional reaction is predicted by the intensity of co-emerging sensations). The importance of this point is that a reaction is not a consequence of a stimulus, which is why both sensitization and desensitization can occur in the absence of a stimulus.
• If interoceptive change has occurred in the intended direction following a response, that response is reinforced (i.e., an avoidant reaction that decreases unpleasant body sensation leads to future avoidance, and a craving reaction that increases pleasant body sensations leads to increased attachment and craving).
• In disequilibrium state, the processing of cognitive-evaluative and learned behavioral output is overemphasized, while awareness of sensory information, including co-emergent interoception, is depleted (i.e., judging and reacting emotionally increase at the cost of one’s normal ability to experience the senses, including co-emerging body sensations).

• This pattern of processing information can be learned with sufficient repetition and become the ‘normal’ state of the system (one becomes over-judgmental and over-reactive), increasing the probability of mental health complications. A chronic emotional disorder is the consequence and representation of learned disequilibrium. This is correlated with impaired insula function and salience network disruption in mental health disorders.
• Co-emergent encoding assists survival by decreasing the probability of memory decay and permits successful memory retrieval of what is perceived as personally important. ‘Relapsing’ into a distressing episode involves the reactivation of a past disequilibrium state from memory, and reconsolidation of the disorder’s memory trace. Recurrent relapses are conceptualized as a consequence of being unable to extinguish the co-emergent memory trace of the disorder.
• Neutralizing reactivity to interoceptive cues by means of equanimity improves therapy outcome (i.e., being aware of body sensations while preventing reactivity during exposure tasks is a more efficacious way of extinguishing a response than focusing on the stimulus alone).
• Therapies that are more equipped to re-establish an equilibrium state are likely to have a greater transdiagnostic applicability (i.e., therapies that help patients neutralize interoceptive conditioning and prevent identifying with value-based judgmental thoughts are more efficacious across a range of disorders than therapies that do not).

In summary, we propose that the basis for the maintenance of numerous psychopathologies is the autonomic reconsolidation of their memory trace through an over-identification with co-emerging interoceptive experience, which renders the extinguishment of the response difficult. This is conceptualized through the CMR as a disequilibrium state between interactive functions of information processing. We suggest that mindfulness training is a skillset that can be used across disorders to recreate equilibrium between functions.

The CMR provides a theoretical rationale for the use of mindfulness practice across a broad range of disorders. Although controlled studies provide support for this view, the practical advantage of using this model in clinical practice can only be ascertained through additional comparisons of therapies that promote interoceptive exposure with those that do not. Future studies are also needed to specifically investigate the role of mindfulness in affecting memory reconsolidation associated with mental health disorders. Moreover, studies are needed to clarify the possibility that interoceptive exposure alone is most helpful to patients who are most concerned about the physical consequences of obvious somatic sensations (e.g., panic symptoms, pain) compared to other consequences (e.g., fear of failure). As it is done in MiCBT, for example, interoceptive desensitization is initially part of mindfulness meditation and followed by combined interoceptive and situational exposure methods to achieve desensitization transdiagnostically. The clinical field would also benefit from studies examining the possibility that interoceptive desensitization facilitate situational exposure, as it is found in MiCBT.

Using the CMR can greatly simplify and facilitate the case-conceptualization of a clinical condition and assist in guiding the direction of therapy. According to the CMR, since the locus of reinforcement is interoception, using interoceptive desensitization transdiagnostically is likely to yield good therapy outcomes across a wide range of disorders. Mindfulness-based techniques of body scanning that include equanimity promote interoceptive desensitization and decrease the propensity for emotional reactivity. The extant literature indicates that they are also more likely to access and neutralize the emotional load of cognitive schemas and help prevent relapse. Thus, using interventions which integrate interoceptive desensitization frees the clinician from the limitations posed by diagnostic-specific therapies, and redirects attention to the patients’ disequilibrium state and to the best possible ways of reducing its effects through equanimity.

## Author Contributions

Both authors listed have made a substantial, direct and intellectual contribution to the work, and approved it for publication.

## Conflict of Interest

Both authors have been paid for developing and delivering educational presentations and workshops for the MiCBT Institute, Australia.
